# Multifunctional Organic Molecule for Defect Passivation of Perovskite for High-Performance Indoor Solar Cells

**DOI:** 10.3390/ma18010179

**Published:** 2025-01-03

**Authors:** Chenqing Tian, Dongxue Liu, Yixin Dong, Yajie Wang, Tinghuan Yang, Yang Yang, Meng Zhang, Erxin Zhao, Nan Wu, Zheng Zhang, Ye Yang, Yongshuai Gong, Buyi Yan, Shengxiong Zhang, Lu Zhang, Tianqi Niu

**Affiliations:** 1 Key Laboratory of Applied Surface and Colloid Chemistry, National Ministry of Education, Shaanxi Key Laboratory for Advanced Energy Devices, Shaanxi Engineering Laboratory for Advanced Energy Technology, School of Materials Science and Engineering, Shaanxi Normal University, Xi’an 710119, China; chenqing_tian@snnu.edu.cn (C.T.); yajie_wang1999@snnu.edu.cn (Y.W.); th_yang@snnu.edu.cn (T.Y.); yy20221014@snnu.edu.cn (Y.Y.); meng_zhang@snnu.edu.cn (M.Z.); erxinzhao@snnu.edu.cn (E.Z.); wunan@snnu.edu.cn (N.W.); 1zhengzhang@snnu.edu.cn (Z.Z.); yeyang@snnu.edu.cn (Y.Y.); 2 Three Gorges Corporation, Science and Technology Research Institute, Beijing 101199, China; dong_yixin@ctg.com.cn (Y.D.);; 3 Microquanta Semiconductor Co., Ltd., Hangzhou 310027, China

**Keywords:** perovskite solar cells, defect passivation, indoor photovoltaic, low-light conditions

## Abstract

Perovskite solar cells (PSCs) can utilize the residual photons from indoor light and continuously supplement the energy supply for low-power electron devices, thereby showing the great potential for sustainable energy ecosystems. However, the solution-processed perovskites suffer from serious defect stacking within crystal lattices, compromising the low-light efficiency and operational stability. In this study, we designed a multifunctional organometallic salt named sodium sulfanilate (4-ABS), containing both electron-donating amine and sulfonic acid groups to effectively passivate the positively-charged defects, like under-coordinated Pb ions and iodine vacancies. The strong chemical coordination of 4-ABS with the octahedra framework can further regulate the crystallization kinetics of perovskite, facilitating the enlarged crystal sizes with mitigated grain boundaries within films. The synergistic optimization effects on trap suppression and crystallization modulation upon 4-ABS addition can reduce energy loss and mitigate ionic migration under low-light conditions. As a result, the optimized device demonstrated an improved power conversion efficiency from 22.48% to 24.34% and achieved an impressive efficiency of 41.11% under 1000 lux weak light conditions. This research provides an effective defect modulation strategy for synergistically boosting the device efficiency under standard and weak light irradiations.

## 1. Introduction

With the rapid development of the Internet of Things (IoTs), the operation of ecosystems through wireless networks requires a persistent and portable power supply to facilitate the terminal integration and interaction of various electronic devices [1]. In practical application scenarios, such as indoor lighting, cloudy days, and dawn/dusk periods, electronics frequently experience lower light intensities [2]. Indoor photovoltaics can convert the photonics absorbed from common indoor light sources into electrical power, endowing them with a promising solution for efficient power generation in IoTs systems [3]. Among the indoor photovoltaic technologies, PSCs have garnered widespread research interest in recent years due to their excellent optoelectronic properties, low production cost, and solution-processing compatibility. Compared to traditional silicon solar cells with a lower bandgap of 1.1 eV [4,5], PSCs exhibit higher efficiency under low-light environments thanks to their unique tunable bandgap and excellent light absorption capabilities [6,7]. Therefore, PSCs demonstrate greater potential and competitive advantages in low-light, indoor photovoltaics applications.

For common indoor light sources, such as LEDs and fluorescent lamps, their emission spectrum typically falls within a narrow range of 400–700 nm [8]. Consequently, wide-bandgap perovskites (bandgap of 1.7–1.9 eV), with well-matched optical absorption within this range, have been widely adopted as absorber layers for indoor photovoltaics [9]. Despite their outstanding optical harvesting under low-light conditions, wide-bandgap PSCs still face several critical challenges, including phase segregation, low activation energy of ionic migration, and lattice distortion, which result in a significant voltage deficit and fragile environment durability under light or humidity stimulus [10,11]. In contrast, the advanced perovskite systems with middle bandgap occupy simple composition structures and well-controlled crystalline routes, facilitating better compositional homogeneity and reduced energy loss in devices. Additionally, the facile preparation procedures allow for the production of these perovskite categories that are highly compatible with scalable manufacturing processes. This enables seamless integration into commercial production lines, ultimately decreasing overall costs. Therefore, the enhancement in compatibility of PSCs for dual-application scenarios in indoor and outdoor lighting conditions is crucial for maximizing their market potential and broadening their application fields.

Under low-light conditions with a smaller carrier yield, the primary factors limiting the performance of PSCs are crystal structural defects and surface imperfections. These defects can capture the photogenerated carriers and elevate the charge transport barrier, resulting in deteriorated non-radiative recombination in devices [12]. As such, effective control of defect distribution within the perovskite materials has become a critical focus for enhancing the performance of PSCs in low-light conditions. For example, Wang et al. adopted a molecular sieve strategy by incorporating the organic small molecule phthalimide (2-N) into the antisolvent, effectively regulating the crystallization quality of perovskite and achieving an indoor power conversion efficiency (PCE) of 40.07% under 1062 lux light conditions [13]. Heet al. incorporated guanidine iodide and used 2-(4-methoxyphenyl)ethylammonium bromide (CH_3_O-PEABr) to passivate the surface of perovskite films, achieving a power conversion efficiency (PCE) of 40.1% for perovskite-based indoor photovoltaics (PIPVs) [14]. Moreover, Ma et al. employed oleylammonium iodide in trichloromethane solvent as a dual additive to modify the wide-bandgap perovskite precursor, effectively reducing grain boundary and bulk defects and achieving a PCE of 44.72% under 1000 lux light conditions [15]. Functional organic modifiers in bulk perovskites can passivate charged defects through chemical coordination, ionic bonding, or hydrogen interaction, thereby mitigating their adverse effects on carrier extraction efficiency [16]. However, faced with the complexity of defect categories and spatial distribution within the solution-processed perovskite films, the singular targeted molecules with limited defect capture ability make it difficult to meet the comprehensive passivation requirement. Although the increased incorporation types can introduce more functional moieties and strengthen the final electrical doping effects, this inevitably elevates fabrication costs and complicates trial-and-error procedures, thereby hindering scalability and practical production. Hence, developing functionalized molecule structures with synergistic passivation effect is crucial for the high efficiency of indoor PSCs.

In this work, we propose an effective trap passivation strategy by using p-aminobenzenesulfonic acid sodium salt (4-ABS) as the multifunctional additive to regulate the crystallization process of perovskite films while effectively passivating film defects. The experimental results demonstrate that the electron-donating amine and sulfonic-acid groups in the 4-ABS structure can serve as Lewis bases to coordinate the under-coordinated Pb^2+^, facilitating the alleviated positively charged defects. The high coordination affinity of 4-ABS with the octahedra framework can further optimize the crystallization kinetics of perovskite, improving morphology quality with mitigated grain boundaries within films. The synergistic optimization effects on trap suppression and crystallization modulation upon 4-ABS addition can reduce energy loss and mitigate ionic migration under low-light conditions. As a result, the optimized device demonstrated enhanced efficiencies of 24.34% and 41.11% under standard and weak lighting conditions, respectively, outperforming the values of 22.48% and 36.19% for the control. Moreover, the unencapsulated PSCs retained almost the original performance after 2000 h of ambient storage.

## 2. Materials and Methods

### 2.1. Materials

Sodium 4-aminobenzenesulfonate (4-ABS) was bought from Bide Pharmatech Co., Ltd. (Shanghai, China). Formamidine iodide (FAI, 99.95%), methylammonium iodide (MAI, 99.5%), methylammonium chloride (MACl, 99.5%), Pb (II) iodide (PbI_2_, 99.99%), and Spiro-OMeTAD were purchased from Advanced Election Technology Co., Ltd. (Liaoning, China). Phenylethylammonium iodide (PEAI, 99.5%) was purchased from Xi’an Yuri Solar Co., Ltd. (Xi’an, China). N, N-dimethylformamide (DMF) (anhydrous, 99.8%) and dimethyl sulfoxide (DMSO) (anhydrous, ≥99.9%) were obtained from Alfa Aesar Inc. (Tewksbury, MA, USA). 4-tert-butylpyridine (t-BP, 99%) and lithium bis(trifluoromethylsulfonyl)imide salt (LiTFSI) were purchased from Sigma-Aldrich (St. Louis, MO, USA). Chlorobenzene (CB) (anhydrous, 99.8%) and isopropanol (IPA) (anhydrous, 99.5%) were purchased from Sinopharm Chemical Reagent Co., Ltd. (Shanghai, China). All chemicals and solvents were utilized as received without further purification.

### 2.2. Perovskite Film Preparation and Device Fabrication

Solution preparation: The solution was prepared under an inert atmosphere inside a nitrogen glove box. A 1.3 M FA_0.92_MA_0.08_PbI_3_ perovskite precursor solution with extra 40 mol% MACl doping was prepared by dissolving PbI_2_, FAI, MAI, and MACl in a 1 mL mixture of DMF and DMSO (in a 4:1 volume ratio) and stirring overnight. Different concentrations of 4-ABS were added to the precursor solution as a dopant. The perovskite solutions were filtered before solution casting. The PEAI was dissolved in isopropyl alcohol (IPA) at a concentration of 5 mg/mL and heated at 70 °C for 5 min prior to solution casting. The Spiro-OMeTAD solution was prepared by dissolving 90 mg of Spiro-OMeTAD, 22 μL of lithium bis(trifluoromethanesulfonyl) imide in acetonitrile, and 36 μL of 4-tert-butylpyridine in 1 mL of chlorobenzene.

Device fabrication: FTO-coated glass (2.5 × 2.5 cm^2^) was cleaned by sequential sonication procedure using Hellmanex III detergent (Merck KGaA, Darmstadt, Germany) and water for 30 min to remove the surface impurities and dried under N_2_ flow, followed by ozone plasma for 20 min. The c-TiO_2_ layer was prepared by chemical bath deposition with the FTO substrate immersed in a TiCl_4_ (CP, Sinopharm Chemical Reagent Co., Ltd.) aqueous solution with a volume ratio of TiCl_4_:H_2_O equal to 0.0225:1 at 70 °C for 1 h. These substrates were subsequently annealed at 200 °C for 30 min and UV-treated for 15 min before perovskite preparation. During the spin-coating procedure of perovskite, 70 μL of perovskite solution was spread over the substrate at 6000 rpm for 30 s. At 15 s before the end of the procedure, 100 μL diethyl ether was dropped onto the substrate. The substrates were then dried on a hotplate at 100 °C for 30 min. After cooling the perovskite film to room temperature, 45 μL of PEAI solution was dropped on the perovskite film during a spin-coating procedure at 4000 rpm for 30 s, followed by thermal annealing at 100 °C for 5 min. Subsequently, the hole transporting layer was deposited by spin coating the Spiro-OMeTAD solution at 5000 rpm for 30 s. Finally, a gold electrode (80 nm) was thermally evaporated under vacuum, and PSCs were oxidized for 24 h before testing.

## 3. Results and Discussion

### 3.1. 4-ABS–Perovskite Interactions

**Figure 1:**
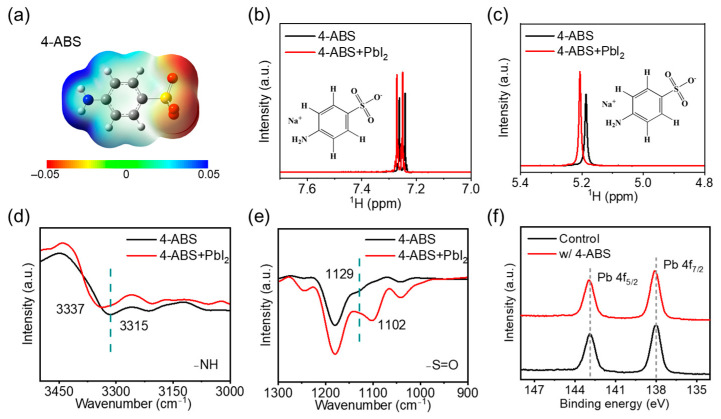
(**a**) ESP distribution of 4-ABS. (**b**,**c**) ^1^H NMR spectra of pristine 4-ABS and 4-ABS + PbI_2_ mixed solutions. (**d**,**e**) FTIR spectra of 4-ABS and 4-ABS + PbI_2_ films. (**f**) XPS spectra of Pb 4f signal in high-resolution XPS spectra for the control and 4-ABS-based perovskite films.

Under low-light conditions with lower carrier generation, solar cells require efficient defect suppression to maintain high performance, which is even more urgent than the standard illumination scenarios [17]. The 4-ABS molecule, equipped with functional groups such as amino and sulfonic acid groups, presents a promising candidate for defect passivation studies (Figure 1a). These functional moieties exhibit strong potential for interaction with the perovskite framework, enabling targeted passivation of specific defect types and facilitating precise defect management [18,19,20,21]. Electrostatic potential (ESP) calculations revealed a distinct electronegative nature around the sulfonic acid group and a strong positive potential near the amino group, highlighting the chemical versatility of 4-ABS. Specifically, the oxygen atoms within the sulfonic acid group may coordinate with undercoordinated Pb^2+^ ions, while the ammonium group would form the hydrogen bonding with iodides from octahedra lattices and further support the lone pair of electrons to passivate the positively charged defects. The ionized Na^+^ from the organometallic salt can serve as the interstitial doping to passivate the anion vacancies in the perovskite lattices [22]. This multi-site interaction suggests that 4-ABS can effectively mitigate charge recombination centers within the perovskite lattice and at grain boundaries. Moreover, the strong chemical coordination of 4-ABS with the perovskite framework would facilitate its regulation of the crystallization kinetics of perovskite and optimize the film quality.

The chemical interactions between 4-ABS and perovskite were evaluated by the proton nuclear magnetic resonance (^1^H NMR), Fourier infrared spectroscopy (FTIR), and X-ray photoelectron spectroscopy (XPS) measurements, respectively. As shown in Figure 1b,c, compared to pure 4-ABS, the proton signals on the amino group and the aromatic ring in the mixture exhibited a significant downfield shift. This change can be attributed to the decreased electron density around the hydrogen nuclei in 4-ABS, suggesting the formation of hydrogen bonding with PbI_2_. Further analysis indicated the Pb-I inorganic framework of the perovskite may engage in strong coordination with the amino and sulfonate groups of 4-ABS, significantly altering the molecular environment. The FTIR spectra were then determined on the pure 4-ABS films with and without PbI_2_ addition (Figure 1d,e). When 4-ABS was mixed with the equimolar ratio of PbI_2_, the stretching vibration of the N-H bond in the amino group shifted from 3315 to 3337 cm^−1^, suggesting the formation of hydrogen bonds between the amino group and the I^−^ ions. Additionally, the stretching vibration of the S=O bond in the sulfonate group of perovskite films shifted from 1129 to 1102 cm^−1^ upon the 4-ABS addition, illustrating the coordination interaction between the sulfonate group and the vacant orbitals of Pb^2+^. These changes in vibrational modes provide further evidence for the strong interactions between 4-ABS and the Pb-I inorganic framework, which involve a synergistic manner of hydrogen interaction and coordination bonding between the 4-ABS and perovskite. Furthermore, X-ray photoelectron spectroscopy (XPS) analysis demonstrated a 0.1 eV shift towards lower binding energy of the Pb 4f signals upon the introduction of 4-ABS (Figure 1f) [23,24,25], which suggested the formation of strong chemical interactions between the Pb–I inorganic framework and the functional moieties of 4-ABS. Such interactions are likely driven by the coordination of the sulfonic and amino groups within 4-ABS with undercoordinated Pb^2+^ ions, providing direct evidence for defect passivation at the atomic level. These findings highlight the critical role of 4-ABS in enhancing the structural integrity and electronic quality of the perovskite system, which offers direct molecular-level insights into the synergistic passivation effects of 4-ABS on perovskites.

### 3.2. Film Characterizations

**Figure 2:**
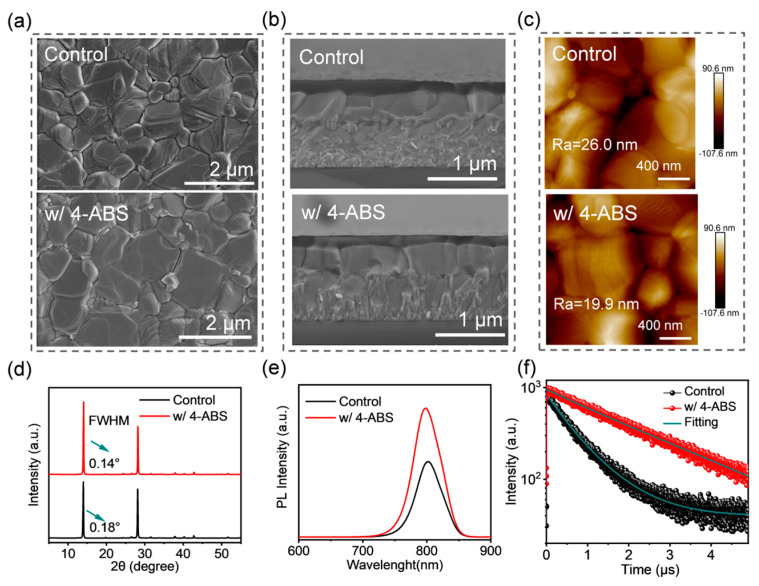
(**a**) Top-view SEM images of control and 4-ABS-based perovskite films. (**b**) Cross-sectional SEM images of whole devices. (**c**) AFM images of control and 4-ABS-based perovskite films. (**d**) XRD patterns. (**e**) Steady-state PL spectra. (**f**) TRPL spectra of control and 4-ABS-based perovskite films.

Figure 2a presents the top-view scanning electron microscopy (SEM) images of the control and 4-ABS-based perovskite films, offering a direct comparison of the morphological quality. Compared to the control, the perovskite grains with 4-ABS incorporation exhibited more regular alignment, tighter inter-grain connections, and significantly larger sizes. The introduction of 4-ABS led to a marked improvement in the crystallinity of the perovskite film, which contributed to the reduced density of grain boundary on the film surface and improved the overall structural integrity of films. As shown in Figure 2b, cross-sectional SEM images of the control and optimized samples showed distinct differences in the stacking quality of perovskite crystals within devices. In the control sample, the cross-sectional image displays disordered grain arrangement and uneven grain size distribution, indicating the presence of non-uniform nucleation and competitive grain growth during film formation. Encouragingly, the optimized sample exhibited well-connected perovskite grains with a uniform size distribution. Such morphological optimization not only reduces the defect density within bulk films but also promotes more efficient diffusion and transport of photogenerated carriers, leading to a significant enhancement in the optoelectronic performance of the device. Further structural analysis confirmed the negligible variation in film thickness (ca. 420 nm) for the control and 4-ABS cases. To assess the changes in surface topography, atomic force microscopy (AFM) was then employed. Figure 2c shows the average root mean square roughness (Ra) of the perovskite films was reduced from 26.0 to 19.9 nm with the addition of 4-ABS. The decrease in surface roughness facilitates more intact contact between the perovskite and the upper hole transport layer (HTL), which would reduce the interfacial resistance and improve the hole extraction at the anode interface.

We then employed X-ray diffraction (XRD) to investigate the impact of 4-ABS on the crystallinity of the perovskite films. As shown in Figure 2d, the perovskite films exhibited two prominent diffraction peaks at 14.0° and 28.0°, corresponding to the (110) and (220) crystal planes, respectively. The absence of the peak shift in the 4-ABS film illustrated that the ionic compositions from the dopant did not incorporate into the perovskite lattices. In contrast, the 4-ABS-based sample showed a significant enhancement in the intensity of the (110) diffraction peak compared to the control case, indicating improvements in the orientation order and crystallinity of the perovskite. Additionally, the full width at half maximum (FWHM) derived from the (110) diffraction peak in the optimized sample was noticeably reduced, indicating an increase in grain sizes. These findings provide direct evidence for the role of 4-ABS in modulating the crystalline quality of the perovskite films.

To further investigate the effect of 4-ABS on the optical properties of perovskite films, ultraviolet–visible (UV–vis) absorption and photoluminescence (PL) spectroscopy measurements were conducted. As detailed in Figure S1 (Supplementary Information), the absorption edge of perovskite films with 4-ABS does not exhibit a significant shift, indicating that the optical band gap remains unchanged. Similarly, the steady-state PL spectra (Figure 2e) reveal that the PL peak position of the optimized sample aligns with that of the control sample, which is consistent with the UV–vis absorption results and confirms that the introduction of 4-ABS does not affect the optical bandgap of the perovskite. In contrast, the 4-ABS-based film shows a notable enhancement in PL intensity, with a blueshift of peak position compared to the control sample. This suggests that 4-ABS effectively suppresses the trap-assisted non-radiative recombination loss in perovskite films.

To further investigate the carrier dynamics, time-resolved fluorescence (TRPL) spectroscopy was conducted to measure the carrier lifetime in the control and 4-ABS-treated perovskite films. The experimental data were fitted using a biexponential decay function, which allows for the extraction of carrier lifetime values [26,27]:

(1) $$\tau \left(t\right)=A_{1}e^{-(t-t_{0})/\tau_{1}}+A_{2}e^{-(t-t_{0})/\tau_{2}}$$

where *A*_1_ and *A*_2_ represent the relative amplitudes, while *τ*_1_ and *τ*_2_ denote the fast and slow decay times, respectively. The *τ*_1_ is primarily associated with defect-induced non-radiative recombination processes, whereas the *τ*_2_ reflects the radiative recombination of charge carriers. Figure 2f presents the TRPL decay curves for the perovskite films, and the detailed fitting parameters are listed in Table S1. From the fitting results, the average carrier lifetime (*τ_ave_*) of the control film was calculated to be 0.77 μs, whereas the perovskite film incorporating 4-ABS exhibited a significantly increased carrier lifetime of 2.87 μs. This remarkable increase in the *τ_ave_* corresponds to a substantial enhancement in the charge transport efficiency of the film [28,29]. These results provide strong evidence that the incorporation of 4-ABS improves the crystallinity and morphology of the perovskite films but also significantly enhances the carrier dynamic properties by effectively suppressing charged defects.

### 3.3. In Situ Investigation into Crystallization Dynamics

**Figure 3:**
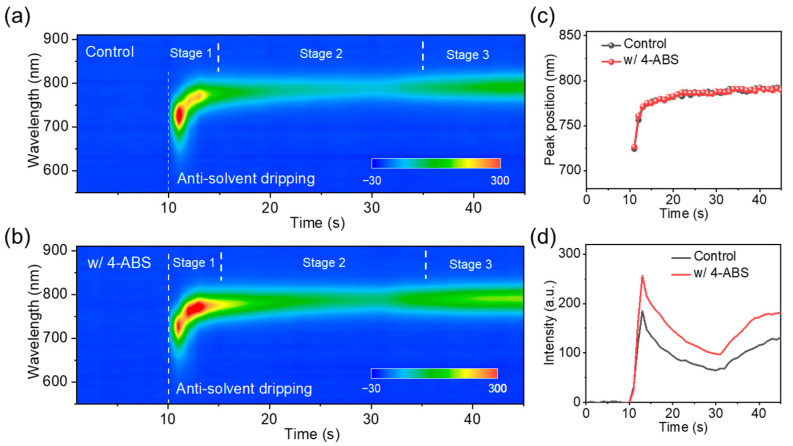
(**a**,**b**) In situ PL spectra of films with or without 4-ABS during the spin-coating process. (**c**) Dynamic evolution of PL intensity at 760 nm for the perovskite films. (**d**) Dynamic evolution of PL peak position of perovskite films.

To gain deeper insights into the impact of 4-ABS on defect stacking during film formation, we performed real-time in situ photoluminescence (PL) spectroscopy to monitor the film deposition process. Figure 3a,b show the in-situ PL spectra of the films during the spin-coating stage, while the extracted PL peak positions and intensity evolution at 760 nm are presented in Figure 3c and Figure 3d, respectively. Three primary transformation stages were detected during the crystallization process.

Stage I: The initial crystallization of the α-phase perovskite occurred around 10 s, coinciding with the moment when the antisolvent was dripped. At this point, the PL peak exhibited a slight redshift from approximately 760 nm to 790 nm (Figure 3c). This shift could be attributed to two factors: (i) the preferential crystallization of FA cations over MA and Cs cations on the surface and (ii) the reduction in quantum confinement effects as the crystal size increased during growth [30]. Within 10 to 15 s, the PL intensity significantly increased, indicating the increased nucleation density of the perovskite on the film surface upon antisolvent dripping [31,32]. Stage II: During the period from ca. 15 to 35 s, the PL intensity of α-FAPbI_3_ decreased notably (Figure 3a,b). This reduction can be attributed to solvent evaporation and film recrystallization [33,34]. Stage III: After 35 s, the PL intensity gradually increased and eventually stabilized, indicating the completion of the film crystallization process.

Notably, although the PL peak positions were consistent between the control and 4-ABS samples, the PL intensity of the control film was significantly lower than that of the modified sample during Stage II. This suggests severe defect accumulation in the control sample during crystallization. These observations demonstrate that the addition of 4-ABS effectively facilitated defect passivation in perovskite films. Specifically, 4-ABS enabled synergistic passivation of under-coordinated Pb^2+^ and iodine vacancies, allowing in situ crystallization optimization and defect passivation during film formation.

### 3.4. Electrical Properties of Devices

**Figure 4:**
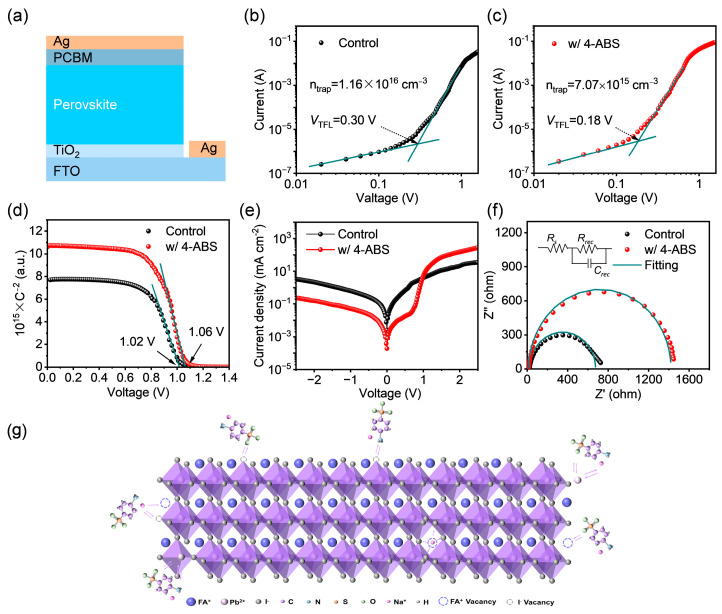
(**a**) Schematic diagram of the device structure for dark *I-V* measurement. (**b**,**c**) Dark *I-V* measurement of the electron-only device. (**d**) Mott–Schottky plots. (**e**) Dark *J–V* curves. (**f**) Nyquist plots of the control and with 4-ABS perovskite solar cells. (**g**) Schematic diagram of 4-ABS interaction with perovskite.

To further investigate the effect of 4-ABS on the electrical properties of the perovskite, we constructed an electron-only device consisting of FTO/c-TiO_2_/perovskite/PCBM/Ag. The space charge limited current (SCLC) model was employed to quantify the defect density within perovskite films (Figure 4a). The trap density was calculated using the following formula [26]:

(2) $$n_{trap}=\frac{2\varepsilon_{0}\varepsilon_{r}V_{TFL}}{{eL}^{2}}$$

where *V_TFL_* is the trap-filled limit voltage, *L* is the thickness of the perovskite layer (approximately 420 nm), *e* is the electron charge (*e* = 1.6 × 10^−19^ C), *ε*_0_ is the vacuum permittivity (*ε*_0_ = 8.854 × 10^−12^ F/m), and *ε_r_* is the relative permittivity. By fitting the dark *I-V* curves, the trap density of the control sample was calculated to be 1.16 × 10^16^ cm^−3^, while the value of the 4-ABS case was significantly reduced to 7.07 × 10^15^ cm^−3^, indicating the efficient defect passivation by 4-ABS addition (Figure 4b,c). We also performed Mott–Schottky measurements to verify the defect passivation effect of 4-ABS on the built-in electric field (*V_b__i_*) in devices (Figure 4d). After the introduction of 4-ABS, the higher slope extracted from the linear regime illustrated the reduced charge residue at the charge extraction interface. Further, the higher *V_bi_* of the 4-ABS-based device facilitates the strengthened driving force for the carrier transport and extraction, thereby endowing the improvement of *Voc* and *FF* in devices [35].

Dark *J-V* measurements (Figure 4e) were widely utilized to investigate charge accumulation features in devices. Compared to the control device, the 4-ABS device demonstrated a notably reduced dark current and a steeper extraction slope under forward bias conditions. This observation indicates that incorporating 4-ABS effectively improves interfacial charge extraction efficiency and mitigates charge accumulation, underscoring its role in enhancing interfacial carrier kinetics. Furthermore, we conducted electrochemical impedance spectroscopy (EIS) tests on devices in dark conditions (Figure 4f). We fitted the data using the equivalent circuit model and derived the fitted parameters shown in Table S2. After modification with 4-ABS, the series resistance (R_s_) decreased from 25.6 to 16.6 Ω, while the recombination resistance (R_rec_) increased from 650.2 to 1404.0 Ω. The improved transport resistance reflects the lower recombination rate in the 4-ABS device, coinciding with the suppressed trap density and improved charge transport properties with 4-ABS doping [36].

Collecting the observations above, we employed the 4-ABS as a precursor modifier (Figure 4g) to optimize the film growth and suppress the trap sites within perovskite films. The trace amount of salt doping did not alter the crystal structure or phase transition route of the perovskite [37,38]. More importantly, the organic anion and metal cation in the 4-ABS can systematically passivate the anionic and cationic defects within perovskite lattices. The Na^+^ cations with high diffusion capability tend to accumulate within the grain boundaries, serving to passivate the FA^+^ vacancies and form the electrostatic interaction with halide anions to increase the ionic migration barrier of perovskite films [39]. Moreover, the electron-donating amine and sulfonic-acid groups in the sulfanilate structure can serve as Lewis bases to coordinate the undercoordinated Pb^2+^, facilitating the alleviated charged defects and optimized crystalline quality [40]. The synergistic optimizations of 4-ABS contribute to the improved charge transport dynamics and mitigated non-recombination loss in films, which would facilitate the further improvement of device performance.

### 3.5. Photovoltaic Performance

**Figure 5:**
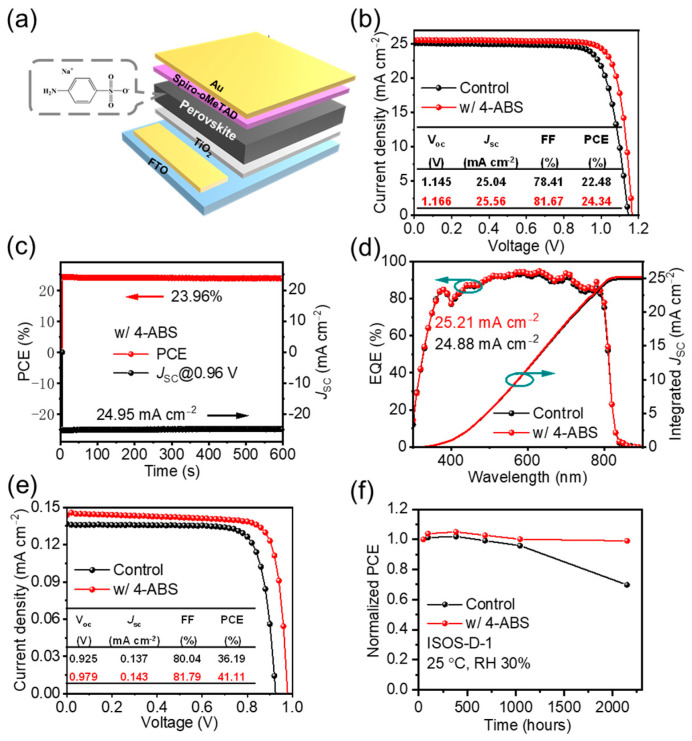
(**a**) Schematic diagram of n-i-p type device structure. (**b**) *J-V* curves of the best-performing control and the 4-ABS devices. (**c**) Stabilized power outputs (SPOs). (**d**) EQE curves and the integrated Jsc for the control and 4-ABS PSCs. (**e**) *J-V* curves of best-performing PSCs under low-light condition (1000 lux). (**f**) The shelf-life stability of PSCs.

To investigate the effect of 4-ABS incorporation on the photovoltaic performance of PSCs, we fabricated devices with the structure FTO/TiO_2_/FA_0.92_MA_0.08_PbI_3_/PEAI/Spiro-OMeTAD/Au, as shown in the schematic diagram in Figure 5a. The control sample achieved a PCE of 22.48%, with J_SC_ = 25.04 mA cm^−2^, *Voc* = 1.145 V, and *FF* = 78.4% (Figure 5b). With the addition of 4-ABS, the device efficiency presented a significant enhancement, mainly derived from the improved *Voc* and *FF*. Under an optimal incorporation concentration of 3 mg/mL, the target device achieved a champion efficiency of 24.34%, with J_SC_ = 25.56 mA cm^−2^, *Voc* = 1.166 V, and *FF* = 81.7%. The steady-state output (SPO) further confirmed the reliability of device performance, showing a steady-state efficiency of 23.96% for the target devices (Figure 5c). Further increased incorporation concentration led to compromised efficiency, which could be attributed to the increased charge recombination centers for energy loss (Figure S2). Additionally, to verify the accuracy of the Jsc in the *J-V* tests, we performed external quantum efficiency (EQE) measurements on both the control and 4-ABS devices. As shown in Figure 5b, both devices exhibited a strong spectral response across a range from 350 nm to 800 nm. The integrated Jsc calculated from the EQE curves was determined to be 24.88 mA cm^−2^ for the control and 25.21 mA cm^−2^ for the 4-ABS sample, which closely matches the *J-V* test results.

Furthermore, it is worth mentioning that the PSCs with 4-ABS exhibited a significantly enhanced efficiency from 36.19% to 41.11% under weak light conditions (1000 lux). This improvement mainly originated from the specific photovoltaic parameters of *Voc* and *FF*, which were increased from 0.925 V to 0.979 V and from 80.04% to 81.8%, respectively. The suppressed trap density and improved carrier transport kinetics can effectively reduce the nonradiative recombination loss and thus contribute to efficiency enhancement. The long-term stability of PSCs was further assessed based on the International Summit on Organic Photovoltaic Stability (ISOS) protocols for dark storage (ISOS-D-1). As shown in Figure 5f, the unencapsulated 4-ABS PSCs retained almost the original performance after 2000 h of ambient storage under ca. 30% humidity conditions. In contrast, the control device degraded to 70% of the initial efficiency during the same duration.

## 4. Conclusions

In this study, we demonstrated an effective trap passivation strategy to alleviate the energy loss and enhance the efficiency of indoor PSCs by introducing multifunctional 4-ABS organometallic salt. The electron-donating amino and sulfonic acid groups in 4-ABS can support the lone pair of electrons to coordinate with the octahedra framework of perovskite and effectively passivate the positively charged defects. The Na^+^ cations tend to accommodate within the grain boundaries, serving to passivate the FA+ vacancies and form the electrostatic interaction with halide anions to increase the structural stability of perovskite films. The synergistic effect of 4-ABS contributed to the enhanced film quality, improved charge transport dynamics, and mitigated nonradiative recombination loss in films. Consequently, the 4-ABS PSCs delivered impressive efficiencies of 24.34% and 41.11% under standard and indoor illumination conditions, respectively. Furthermore, the unencapsulated PSCs demonstrated exceptional stability, which maintained the initial performance after 2000 h of ambient storage. This study provides a facile method to combine the multifunctional modifier with conventional bandgap perovskites to boost the indoor efficiency of PSCs, underscoring the potential for the compatibility of such efficient photovoltaic technology in broad application scenarios.

## Data Availability

The original contributions presented in this study are included in the article and Supplementary Materials. Further inquiries can be directed to the corresponding authors.

## Supplementary Materials

The following supporting information can be downloaded at: https://www.mdpi.com/article/10.3390/ma18010179/s1, Figure S1. UV-visible absorption spectra; Figure S2. J-V curves of devices with 4-ABS added at concentrations of 0–5 mg; Table S1. Fitting parameters for time-resolved fluorescence spectra of control and with 4-ABS; Table S2. Fitting parameters for electrochemical impedance spectra of the control and with 4-ABS.
