# Supplementary material for: Multifunctional Organic Molecule for Defect Passivation of Perovskite for High-Performance Indoor Solar Cells
Source: Materials (Basel). 2025 Jan 3;18(1):179. doi: 10.3390/ma18010179 (PMC11722308; doi:10.3390/ma18010179)
Supplement: Supplementary file 1 [file materials-18-00179-s001.zip › materials-3381313-supplementary/materials-3381313-supplementary.pdf]

Supporting Information

Multifunctional Organic Molecule for Defect Passivation of Perovskite for High-Performance Indoor Solar Cells

Device characterization

The *J-V* curves were collected using a solar simulator (Enlitech, SS-F5-3A) and a Keithley 2400 SourceMeter under 100 mW cm^{-2} . The devices were measured in reverse scans (1.7→0 V, step:0.02 V, delay time: 1 ms) and forward scans (0→1.7 V, step: 0.02 V, delay time: 1 ms). The low-light and low-temperature devices were measured in reverse scans (1.5→0 V, step: 0.02 V, delay time: 1 ms) and forward scans (0→1.5 V, step: 0.02 V, delay time: 1 ms). The area of the cell is 0.09 cm^2 . The lamp's power output was calibrated using a National Renewable Energy Laboratory traceable KG5- filtered silicon reference cell. The external quantum efficiency (EQE) was measured on a QE-R system (Enli Technology Co., Ltd.) using a 300-W Xe lamp as the light source.

Film characterization

X-ray diffraction (XRD) studies were performed using a DX-2700BH diffractometer (Dandong Haoyuan Instrument Co., Ltd.). Steady-state photoluminescence (PL) and time-resolved photoluminescence (TRPL) spectra were recorded with a PicoQuant FT300 spectrometer. The excitation laser wavelength is 510 nm, the frequency is 40 MHz (for PL) and 0.2 MHz (for TRPL). UV-visible absorption spectra were acquired on a PerkinElmer UV Lambda 950 instrument. Grazing incidence wide angle scattering (GIWAXS, λ = 1.24 Å, incidence angle: 0.40°) was performed at the National Facility for Protein Science in Shanghai. The scanning electron microscopy (SEM) images were obtained using a Hitachi SU-8020 field-emission scanning electron microscope (Japan). Atomic force microscopy (AFM) images were obtained by a Bruker Dimension Icon instrument (USA). X-ray photoelectron spectroscopy (XPS) was performed on a photoelectron spectrometer (ESCALAB Xi +, Thermo Fisher Scientific). Fourier transform infrared (FTIR) spectra were recorded with a Bruker VERTEX 70 infrared spectrophotometer using KBr sheets. 1H nuclear magnetic resonance spectra were recorded on a JNM–ECZ400R/S1 apparatus (JEOL, Japan).

Statistical Analysis

Data analysis: Almost all experimental data were analysed and processed using Origin 2023. Normalised case: Among them, long-term stability and thermal stability data were normalized. SEM, XPS, FTIR, UV-Vis, AFM, PL, UPS, J-V, I-T, EQE, C-V were used as original data without normalisation. Other data are obtained by transforming the original data with formulas, which mentioned in the Film characterisation section.

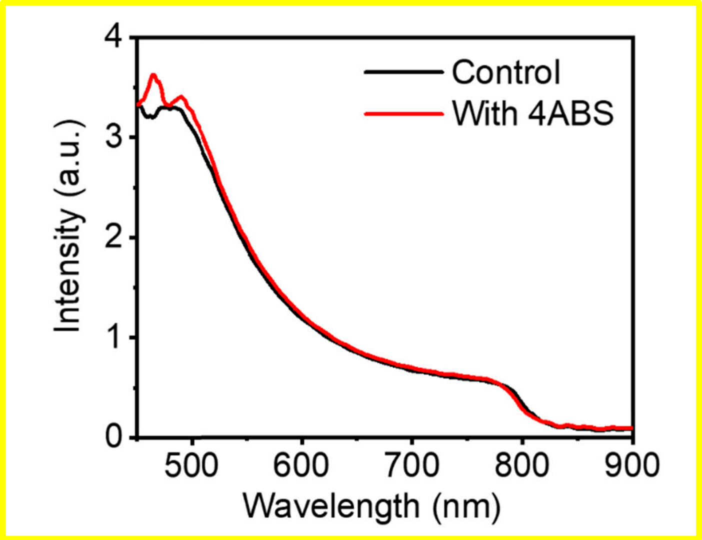

Figure S1. UV-visible absorption spectra.

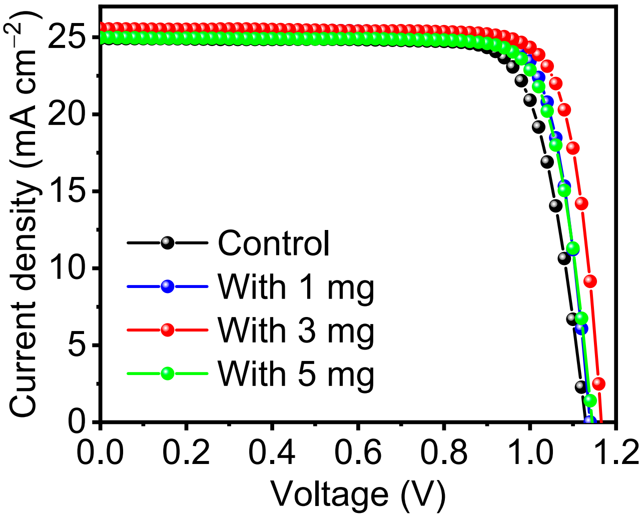

Figure S2. J-V curves of devices with 4-ABS added at concentrations of 0–5 mg.

Table S1. Fitting parameters for time-resolved fluorescence spectra of control and with 4-ABS.

Sample	τ_{ave} (μs)	τ_1 (μs)	A_1 (%)	τ_2 (μs)	A_2 (%)
**Control**	0.77	0.86	78.43	0.43	21.57
**Target**	2.87	3.31	76.53	1.43	23.47

Table S2. Fitting parameters for electrochemical impedance spectra of the control and with 4-ABS.

Sample	R_s (Ω)	R_{rec} (Ω)	C_{rec} (F)
**Control**	25.6	650	1.38×10^{-8}
**With 4-ABS**	16.6	1404	1.21×10^{-8}

Disclaimer/Publisher's Note: The statements, opinions and data contained in all publications are solely those of the individual author(s) and contributor(s) and not of MDPI and/or the editor(s). MDPI and/or the editor(s) disclaim responsibility for any injury to people or property resulting from any ideas, methods, instructions or products referred to in the content.
